# The effect of smart glasses combined with ultrasound on radial arterial catheterization: a randomized controlled trial

**DOI:** 10.1186/s12871-024-02828-8

**Published:** 2024-11-29

**Authors:** Yan Wang, Mingjing Chen, Ting Zou, Yan Weng, Wenjie Mao, Qing Zhong, Haibo Song

**Affiliations:** 1 Department of Anesthesiology, West China Hospital of Sichuan University, Chengdu, 610041 Sichuan P. R. China; 2 Department of Anesthesiology, The People’s Hospital of Jianyang City, Chengdu, 641400 Sichuan P. R. China; 3 Department of Epidemiology, College of Public Health & Health Professions and College of Medicine, University of Florida, Gainesville, FL USA

**Keywords:** Radial arterial, Catheterization, Smart glasses, Ultrasound, Ultrasound-guided interventions, Clinical trial, First puncture success rate, Hand-eye coordination, Satisfaction, Complication rate

## Abstract

**Background:**

The integration of smart glasses with ultrasound technology offers a novel approach to improve the efficiency of radial arterial catheterization. Few studies have investigated the effectiveness of smart glasses in enhancing procedural outcomes in a clinical setting. This study aims to assess whether smart glasses combined with ultrasound can improve the initial success rate of radial artery catheterization compared to traditional ultrasound-guided methods in adults.

**Methods:**

This single-blinded, randomized controlled trial enrolled patients aged 18–70 with American Society of Anesthesiologists physical status I-III, who required radial artery catheterization as part of their procedure under general anesthesia. Patients were randomized 1:1 into the ultrasound group and the smart glasses group. Radial arterial catheterization was carried out by one of six anesthesiologists before general anesthesia. The primary endpoint was the first puncture success rate. Secondary endpoints included hand-eye coordination (measured by head rotations, probe repositioning and needle redirections), operator’s satisfaction.

**Results:**

A total of 222 patients were analyzed, with the smart glasses group demonstrating a higher rate of first puncture success compared to the control group (88.3% [98/111] vs. 72.1% [80/111]; *P* = 0.002; relative risk [RR], 1.23; 95% CI (1.07, 1.40)). Hand-eye coordination improved significantly in the smart glasses group than the control group, including: fewer number of head rotations (0 [0, 0] vs. 3 [2, 6]; *P* < 0.001); fewer number of ultrasound probe repositioning (0 [0, 0] vs. 0 [0, 1]; *P* < 0.001); fewer number of needle redirections (0 [0, 1] vs. 1 [0, 3]; *P* < 0.001). The proportion of positive satisfaction (81 to 100 points) in the smart glasses group was higher (89.2% [99/111] vs. 69.4% [77/111]; *P* <  0.001; RR, 1.29; 95% CI (1.12, 1.48)).

**Conclusions:**

The use of smart glasses significantly improved the first puncture success rate, hand-eye coordination ability and operators’ satisfaction in radial arterial catheterization.

**Trial registration:**

The study was registered at Chictr.org.cn with the number ChiCTR2400081399 on 29/02/2024.

## Introduction

Radial artery catheterization plays a key role in monitoring blood pressure continuously, obtaining frequent blood samples, and analyzing arterial blood gases during surgeries [1]. Ultrasound guidance has significantly enhanced the precision of this procedure [2–4], utilizing techniques such as short-axis out-of-plane (SA-OOP), modified SA-OOP, long-axis in-plane (LA-IP), subcutaneous saline injection, subcutaneous nitroglycerin injection, Dynamic Needle Tip Positioning (DNTP), and laser-assisted ultrasound [5–10]. Advantages of ultrasound guidance include higher success rates, fewer attempts, fewer complications, shorter time to successful intubation, and thus improved patient comfort [3, 11]. However, operators using conventional ultrasound-guided radial artery puncture must frequently adjust their head and viewing position between the patient and the ultrasound screen, which compromises ergonomic efficiency, impedes visualization of the target vessel and proper needle orientation, and may adversely affect the procedure outcomes [12–14]. These issues increase the possibility of repeated puncture, reduce the success of arterial catheterization, and increase the risk of complications [12].

To address these challenges, smart glasses have emerged as a promising technology in medical settings in recent years [12, 15–17]. When combined with ultrasound, smart glasses display virtual ultrasound images directly in the operator’s line of sight, aligning the equipment images and patient in a straight line, eliminating the need for head rotation and improving hand-eye coordination [14]. This helps to maintain the stability of the ultrasound probe and monitor the presence of blood flashback during arterial catheterization [18].

Studies have shown conflicting findings regarding the efficacy of smart glasses in enhancing procedural outcomes. While some studies, like Jang et al. [16] and a recent study reports [18], have reported improved first-attempt success rate and operator satisfaction in pediatric settings, other studies on the use of smart glasses to simulate vascular puncture in adults found no positive effect on success rates, process times, and number of attempts [13, 15, 17]. The impact of smart glasses on success rate is uncertain, and few clinical studies have examined the use of smart glasses combined with ultrasound for this procedure in adults.

This research primarily aimed to assess the impact of integrating smart glasses with ultrasound on enhancing the success rate of radial artery catheterization during the first attempt. Additionally, we assessed hand-eye coordination, the overall number of attempts, overall success rate, ultrasonic localization time, catheterization time, overall complication rate, operator’s satisfaction, and the difficulty level of the procedure. Our hypothesis was that employing smart glasses would improve first puncture success and hand-eye coordination for radial artery catheterization.

## Methods

### Study design

We carried out a randomized controlled trial to investigate whether combining smart glasses with ultrasound for radial artery catheterization in adult patients enhances the first-time puncture success, improves hand-eye coordination, and boosts operator satisfaction. The randomized controlled trial received approval by the Clinical Research Ethics Committee of The People’s Hospital of Jianyang City (NO. JY2024018Z) on February 20, 2024. The trial was registered on the Chinese Clinical Trial Register website at http://www.chictr.org.cn (ChiCTR2400081399; date of registration: February 29, 2024) before enrolling patients. 

### Study setting and participants

The study was performed in The People’s Hospital of Jianyang City (the first hospital of Tertiary Class A comprehensive medicine) from March 2024 to May 2024. The researchers assessed the patients’ eligibility and secured their written consent before the procedure. This article followed the relevant guidelines set out by the CONSORT guidelines for reporting trials. No protocol changes were made after the trial began.

Patients scheduled for general anesthesia, as assessed by the attending anesthesiologist, were included, along with those requiring invasive monitoring of arterial blood pressure or frequent blood sampling for the analysis of gases. Inclusion criteria were an age between 18 and 70 years and ASA physical status of I to III. Patients with pregnancy, peripheral vascular disease, inadequate collateral circulation, recent radial artery puncture, wounds or infections or hematomas at the site of arterial cannulation, hypotension, shock, or cardiac arrhythmia were excluded. On the day of surgery, all eligible patients underwent examination in the preoperative room.

### Randomization and operator

A total of 231 sequentially numbered random envelopes were prepared by a non-clinical member of the research team using a random number generator. Each envelope contained a card with a random number and group information. Participants were randomly assigned in a 1:1 ratio to either the smart glasses group or the ultrasound group (control group). Before the puncture procedure, a trained study nurse opened each envelope. The nurse responsible for group randomization was not involved in patient inclusion or grouping.

All patients in both groups underwent the procedure prior to anesthesia by one of six certified attending anesthesiologists skilled in ultrasound-guided radial artery catheterization (confirmed via written consent through a questionnaire). Each anesthesiologist was right-handed and had extensive experience, having completed exceeding 100 ultrasound-guided arterial catheterizations in adult patients utilizing the short-axis method with the ultrasound probe. All operators had received and passed standard training for smart glasses. Each operator performed at least 26 patients (the same quantity of procedures was performed in each group) and filled out a questionnaire after each procedure. The anesthesiologists did not participate in information collection, measurement, and paper writing.

None of the operators possessed experience in ultrasound-guided arterial cannulation when using a head-mounted display. Prior to the study, the six anesthesiologists were trained in standardized knowledge and acquisition skills. Only the anesthesiologist responsible for ultrasound image acquisition and radial artery insertion were aware of the patient group assignments. However, investigators who measured radial artery depth and diameter and assessed complications and arterial catheter function (during anesthesia, surgery, or resuscitation) were unaware of the group assignments.

### Study procedures

In pre-anesthesia preparation room, each patient received routine monitoring, which included measuring pulse oximetry, conducting an electrocardiogram (ECG), and taking non-invasive blood pressure readings. An intravenous injection of 5 µg of sufentanil was administered before the procedure to alleviate tension and pain associated with arterial catheterization. Following randomization, the operator selected either the left or right radial artery based on the patient’s surgical approach and positioning. During the procedure, the patient lay supine with the wrist gently straightened and secured to a properly sized gauze roll. A Mindray ultrasonic high-frequency linear array probe (L13–3 Ns) was selected. The ultrasonic probe was coated with coupling agent and then tied with sterile protective sleeve. The probe’s depth and gain settings were tailored to each patient, and its position was modified to center the radial artery on the screen. Color doppler ultrasound was utilized to assess vascular patency, vascular abnormalities, and to store short-axis view images prior to obtaining vascular access [11]. After ultrasound scanning of the vessels, which revealed no abnormalities and confirmed blood flow patency, routine sterilization was performed, and local infiltration anesthesia with 1% lidocaine was administered.

**Fig. 1:**
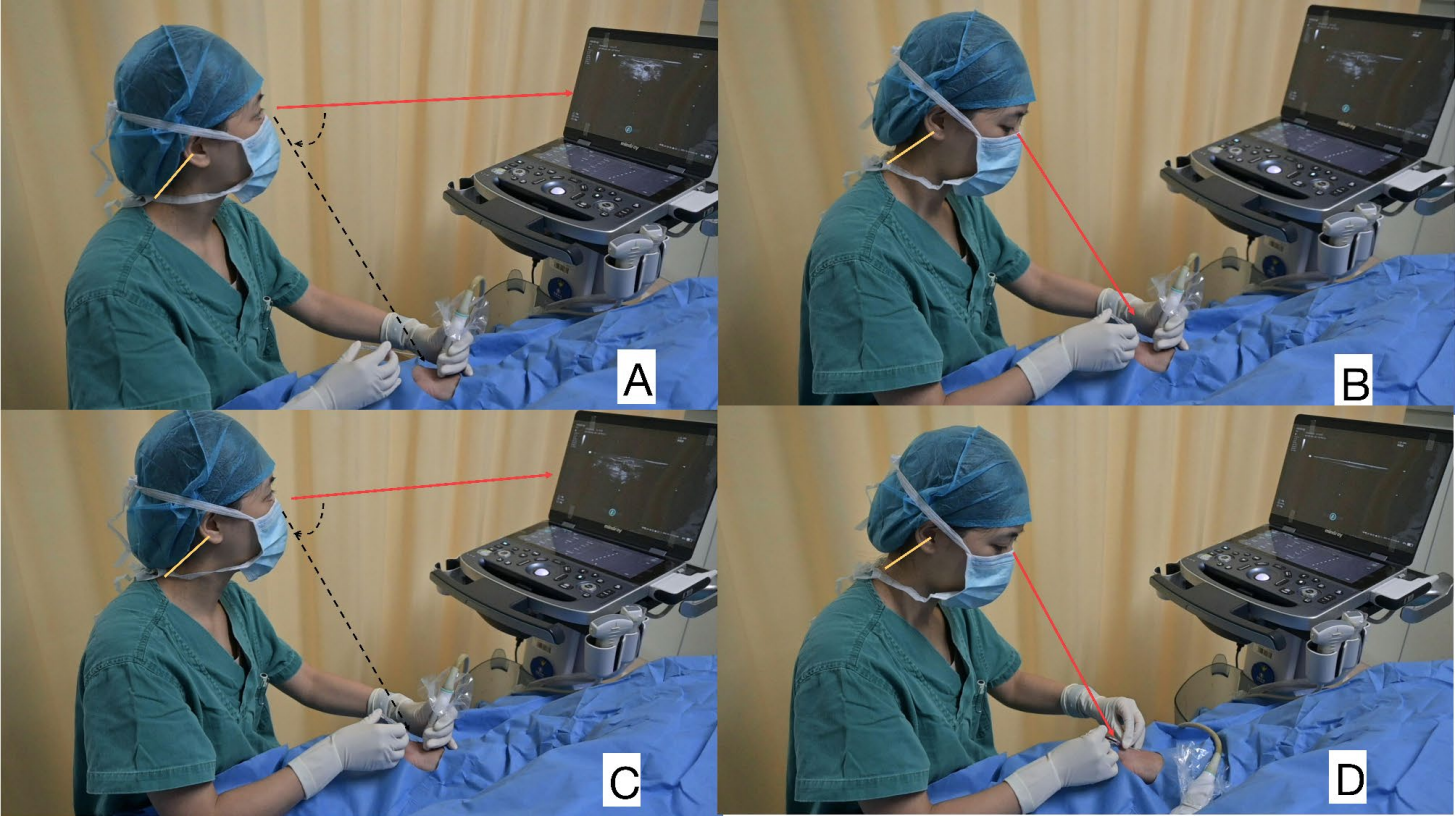
Control group (**A**,** B**, **C**, **D**): the orange lines indicate the operator’s cervical spine, while red lines, black semi-circle and lines illustrate the range of motion and gaze. (**A**) The operator is viewing the ultrasound screen to identify the radial artery. (**B, C**) The operator moves her head, neck, and eyes to switch between the procedure field (cannulation site) and the ultrasound screen during cannulation procedure. (**D**) The operator turns her head to verify unobstructed blood return after needle insertion, confirming successful cannulation

In the ultrasound group (Fig. 1), for standardization and facilitating ease of operation, the ultrasound machine was positioned proximal to the radial artery puncture site on the upper arm side, and the operator was required to sit and use the short-axis out-of-plane (SA-OOP) method to perform the operation [2]. Before inserting the needle, the orientation of the ultrasound probe had to be verified so that the side of the displayed image was aligned with the correct anatomical orientation [11]. The operator held an arterial puncture needle (Introcan-w, 20 G, 1.1*32 mm) in the right hand, the needle and the Angle of the skin were 30 ~ 45 degrees, the intersection point of ultrasound and skin was used as the puncture point, and the tip of the high-echo bright spot was guided to the radial artery lumen under the guidance of ultrasound when the height of the tail of the needle was lowered, the needle was then advanced by 1–2 mm, the blood flow was smooth, and the catheter was inserted and the needle core was withdrawn. The transducer was connected, and the arterial pressure waveform was displayed to confirm the successful tube placement. The catheter was subsequently secured in place using a dressing. If catheter insertion failed after three attempts, or if complications necessitate a change in the puncture site, the procedure was deemed unsuccessful. In such cases, access was attempted in the opposite radial or dorsalis pedis artery. Following this, ultrasound was used to assess the depth and inner diameter of the radial artery and to evaluate any procedural complications, for example, vasospasm or hematoma. Finally, complications during anesthesia and resuscitation after radial artery puncture were recorded in all patients and ductus arteriosus function was assessed.

### Application of smart glasses

**Fig. 2:**
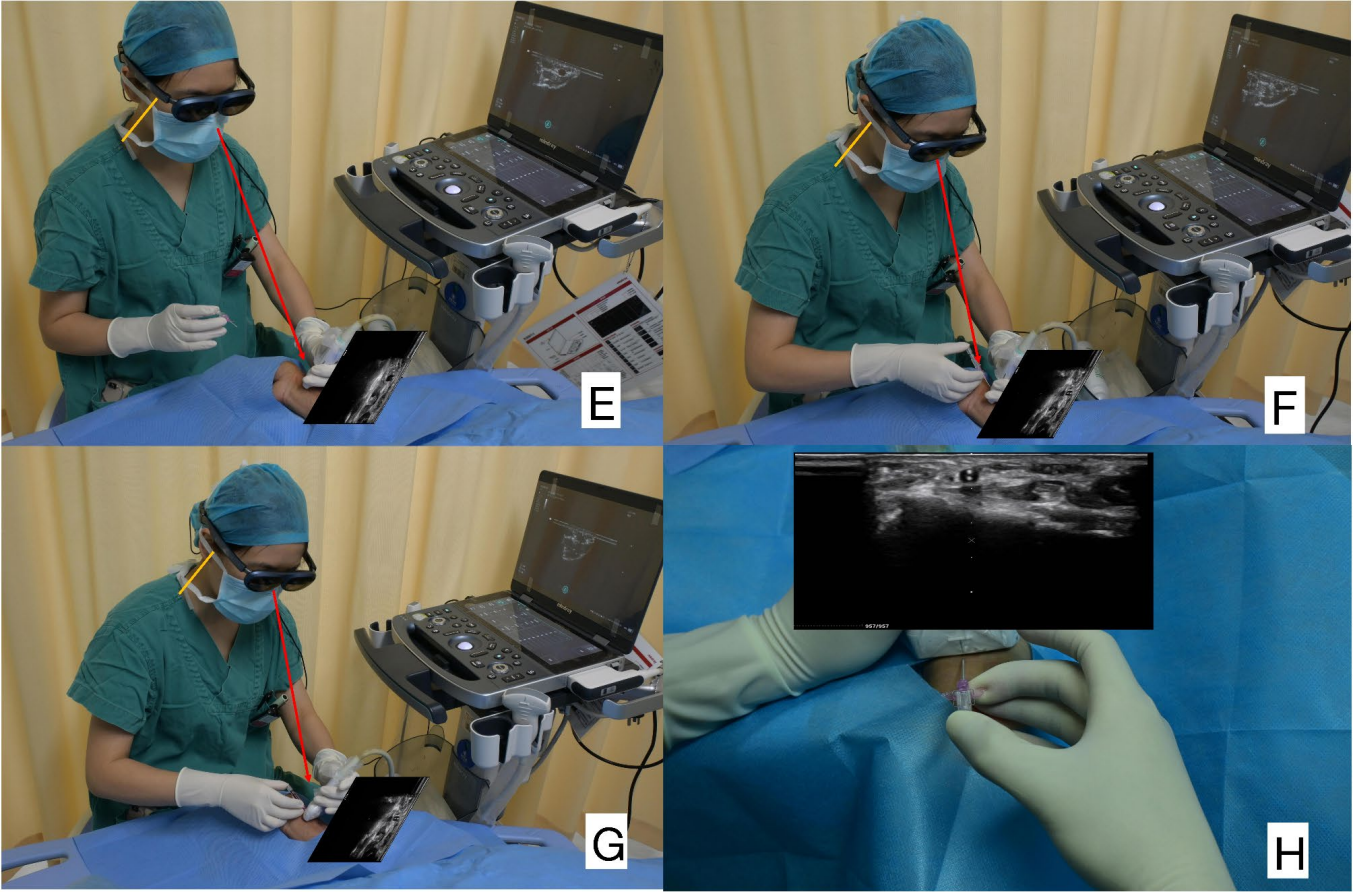
Smart glasses group (**E**, **F**, **G**, **H)**: the orange lines indicate the operator’s cervical spine, and the red lines indicate the range of motion and gaze. (**E, F, G**) With smart glasses, the operator can focus on the ultrasound screen and procedure field (cannulation site) simultaneously without moving her head. (**H**) The ultrasound images were projected directly onto the smart glasses screen within the operator’s visual field

Rokid Max AR smart glasses (RA 201, Hangzhou Ling ban Technology Co., LTD.) served as a device worn on the head for display for the ultrasound machine. The connector box of the smart glasses connected to a high-definition multimedia interface via a digital visual interface, while the interface cable was linked to the left side of the Mindray MX7 device (Mindray Medical Systems) to enable simultaneous display of the ultrasonic screen without any time delay. The smart glasses featured a diopter adjustment knob on the top of the frame, allowed for a range of 0 to 600 diopters. This design made the smart glasses suitable for users who wore corrective lenses. During use, the ultrasound machine continuously supplied power to the smart glasses. In the smart glasses group (Fig. 2), the operator required approximately 5 s to put on and adjust the Smart Glasses prior to the procedure. The procedure of puncture was directly viewed through the gap at the bottom of the display while the ultrasound image was projected onto a smart glasses display worn on the head. The ultrasound machine was positioned on its side, preventing the operator from seeing the ultrasound screen during the procedure, while all other conditions remained consistent with those of the control group. After the operation was completed, the smart glasses were wiped down with disinfectant wipes and secured to the holder for the next use. Each operator was able to complete the procedure successfully [13].

### Outcome measures

The primary outcome was the success rate of the first puncture of the radial artery catheter, defined as a single skin puncture with successful placement of an arterial catheter, which was subsequently verified through a displayed waveform of invasive blood pressure on the cardiac monitor [16].

Secondary outcomes considered in the study were as follows: (1) Hand-eye coordination: include head rotations (defined as head movement greater than 45 degrees, including flexion or rotation), ultrasound probe repositioning (defined as moving the ultrasound probe to recapture the needle or target blood vessel), and number of needle redirections (defined as instances where the operator withdraws the needle, redirects it, or advances it at a different angle). (2) Overall success rate: defined as no more than 3 successful skin piercings in selected radial artery. (3) Overall number of attempts: defined as cumulative count of radial artery puncture. (4) Ultrasonic localization time: defined as the duration from when the ultrasound probe is positioned until the initial skin puncture [18]. (5) Catheterization time: defined as the time interval from the initial skin puncture to the emergence of the invasive blood pressure waveform, independent of the number of arterial catheterizations. (6) The radial artery’s diameter (defined as the inner diameter) and the radial artery’s depth (defined as the vertical distance of the front wall from the surface of the skin). (7) Overall complication rate: include hematoma (defined as low echo area around blood vessel detected on ultrasound), vasospasm (defined as reduction of blood vessel diameter by more than 25% after intubation) [8], artery thrombosis (defined as abnormal echo in blood vessel detected on ultrasound or abnormal circulation at the end of hand), and Catheter malfunction (defined as the inability to monitor or obtain samples despite catheter flushing or changing the dressing) [16]. (8) In order to evaluate the degree of musculoskeletal fatigue and operator satisfaction during operation, we assessed operator’s satisfaction (100 = most satisfied; 0 = dissatisfied) and levels of difficulty (easy, moderate, and difficult) by means of a visual analog scale (VAS) [19]. The scale used an unscaled 10-cm long ruler.

### Statistical analysis

Baseline characteristics were evaluated with the use of various statistical tests, including the independent t-test for two samples, the Mann-Whitney U test, and the Chi-square test. Continuous variables were analyzed either with a two-sample independent t-test or the Mann-Whitney U test, based on their distribution. For categorical variables, the Chi-squared test was employed.

The comparison of the success rate of the initial puncture between the smart glasses group and the control group was conducted using the Chi-squared test. Secondary outcomes with continuous data are tested using the t-test when the data are normally distributed, and Wilcoxon rank-sum test when they are non-normally distributed. Categorical outcomes were evaluated using either Chi-squared tests or Fisher’s exact tests. Kaplan–Meier methods were used to analyze the overall procedure time for successful radial artery cannulation, with group comparisons made through the log-rank test. A significance threshold of α = 0.05 was set for all analyses. Statistical evaluations were conducted using IBM SPSS Statistics version 29 [version 29.0.1.0 (171)].

### Sample size calculation

Based on a literature review, the expected first puncture success rate in the intervention group (using smart glasses combined with ultrasound) was estimated at 90%. This estimation considered a reported first attempt success rate of 87.9% in pediatric under 2 years using smart glasses combined with ultrasound [16], with the understanding that the first puncture success rate in children is generally lower than in adults [9]. The success rate for the control group was approximated at 76%, derived from typical rates found in literature for ultrasound-guided radial artery cannulation [3, 20, 21]. To identify a minimum difference of 14% between the groups with 80% power and a two-sided alpha level of 0.05, it was necessary to include 110 participants in each group. To accommodate a possible 5% dropout, the total sample size was adjusted to 231 participants.

## Results

**Fig. 3:**
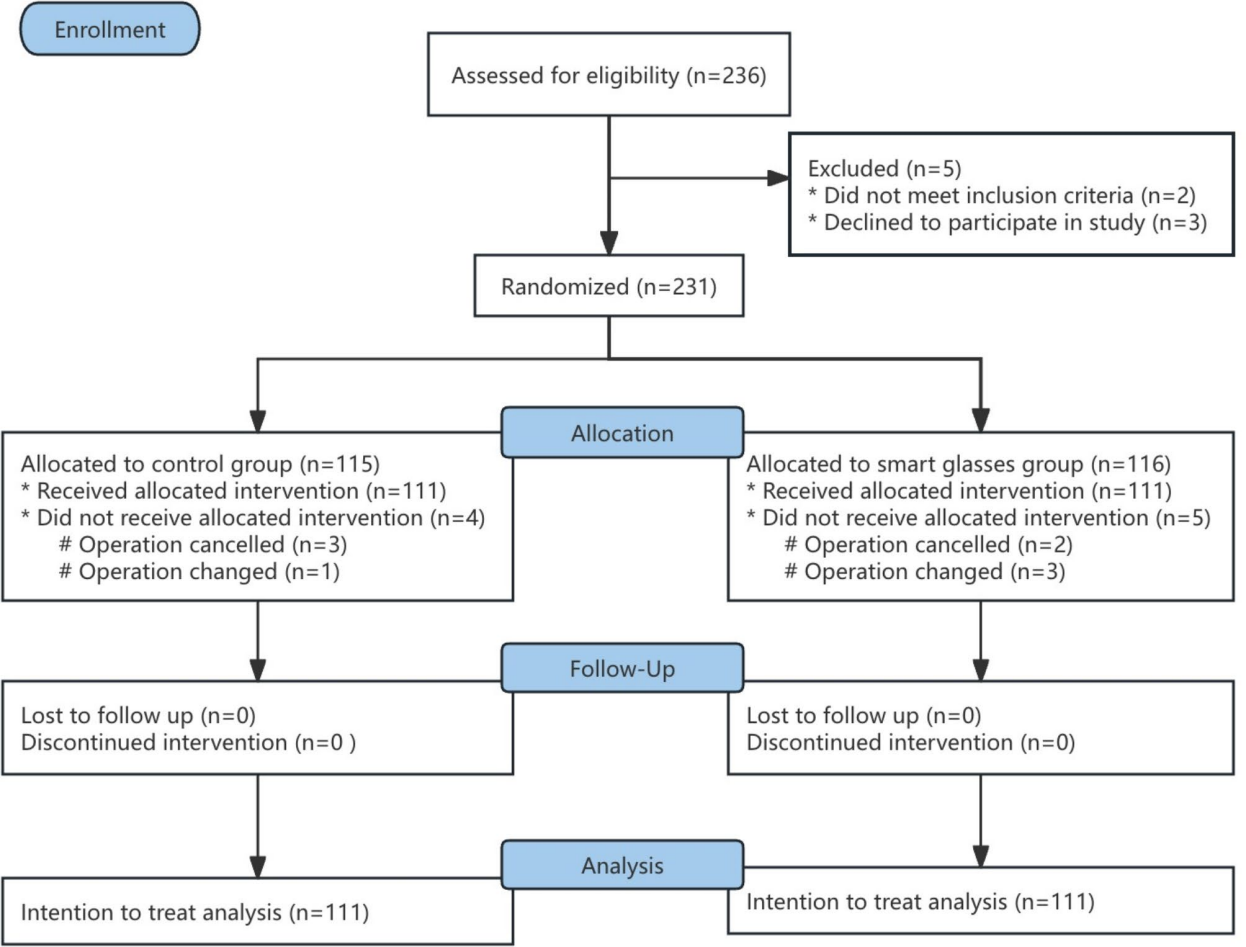
The consolidated standards of reporting trials (CONSORT) flow diagram

The study initially recruited 236 patients. Of these, 5 were excluded: 2 did not satisfy the inclusion criteria, and 3 opted out of participation. Consequently, 231 patients were ultimately included and randomly allocated to either the smart glasses group (*n* = 116) or the ultrasound group (*n* = 115). Nine patients did not undergo the study procedure because the radial artery puncture was canceled by the attending physician due to cancellation of the procedure or a change in the procedure (Fig. 3). Researchers conducted the study in the inpatient operating room of The People’s Hospital of Jianyang City from March to May 2024 and was stopped after full registration of patients. There were no reported violations of the study protocol throughout the study, and no data on primary and secondary outcomes were omitted during the study.

### Baseline characteristics

Baseline characteristics of the 222 subjects included in the final analysis were summarized in Table 1. No substantial differences were observed among patients concerning age, gender, height, weight, body mass index (BMI), puncture site, radial artery diameter and depth, underlying conditions, ASA physical status, or the type of surgery performed.

**Table 1:** Baseline characteristics of patients (*n* = 222)

Characteristic	Control (*n* = 111)	Smart Glasses (*n* = 111)	Standardized difference
Age (y), median [IQR]	56 [49, 60]	54 [49, 61]	0.05
Female, N (%)	62 (55.9%)	61 (55%)	0.02
Height (cm), median [IQR]	158 [154, 164]	158 [155, 165]	− 0.17
Weight (kg), mean ± SD	61.9 ± 9.79	62.9 ± 10.7	− 0.09
BMI, mean ± SD	24.7 ± 3.48	24.8 ± 3.53	− 0.02
Left radial artery, N (%)	62 (55.9%)	58 (52.3%)	0.07
Diameter before cannulation (mm), median [IQR]	2.0 [1.8, 2.3]	2.1 [1.8, 2.4]	− 0.25
Depth before cannulation (mm), median [IQR]	2.4 [1.7, 3.2]	2.3 [1.8, 3.2]	− 0.03
Underlying disease, N (%)			− 0.07
Hypertension	22 (19.8%)	24 (21.6%)	
Coronary heart disease	10 (9%)	10 (9%)	
Diabetes	6 (5.4%)	10 (9%)	
Pulmonary disease	17 (15.3%)	21 (18.9%)	
Arrhythmia	1 (0.9%)	2 (1.8%)	
ASA physical status, N (%)			0
I	29 (26.1%)	30 (27.0%)	
II	68 (61.3%)	66 (59.5%)	
III	14 (12.6%)	15 (13.5%)	
Surgery, N (%)			0
Cardiothoracic Surgery	18 (16.2%)	18 (16.2%)	
Urologic surgery	9 (8.1%)	11 (9.9%)	
General surgery	24 (21.6%)	23 (20.7%)	
Gynecologic surgery	15 (13.5%)	14 (12.6%)	
Orthopedic Surgery	45 (40.5%)	45 (40.5%)	

Standardized difference is the difference in means or proportions divided by the SD and calculates potential imbalance in groups

Continuous variables were expressed as means ± standard deviation, or median [IQR]

Qualitative variables were expressed as n (proportion%). SD = standard deviation

IQR = interquartile range. BMI: body mass index. ASA: American Society of Anesthesiologists

### Primary outcome

The first success rate of radial arterial catheterization was greater in the smart glasses group in comparison to the ultrasound group (88.3% [98/111] vs. 72.1% [80/111]); *P* = 0.002; relative risk [RR], 1.23; 95% CI (1.07, 1.40); Table 2).

**Table 2:** Results of Radial Artery Cannulation in the Control Group and the Smart Glasses Group

Variables	Control (*n* = 111)	Smart Glasses (*n* = 111)	95% CI of Relative Risk or Median Difference	*P* value
*Primary outcome*				
First success rate, N (%)	80 (72.1%)	98 (88.3%)	1.23 (1.07, 1.40)	0.002^d^
*Secondary Outcomes*				
Hand-eye coordination				
Number of head rotations, median [IQR]	3 [2, 6]	0 [0, 0]	-3 (-3, -3)	< 0.001^e^
Number of ultrasound probe repositioning, median [IQR]	0 [0, 1]	0 [0, 0]	0 (0, 0)	< 0.001^e^
Number of needle redirections, median [IQR]	1 [0, 3]	0 [0, 1]	-1 (-1, 0)	< 0.001^e^
Overall success rate, N (%)	103 (92.8%)	109 (98.2%)	1.06 (1.00, 1.12)	0.052^d^
Overall number of attempts, median [IQR]	1 [1, 2]	1 [1, 1]	0 (0, 0)	0.002^e^
Ultrasonic localization time (s), median [IQR]	10 [6, 15]	7 [5, 10]	-2 (-3, -1)	< 0.001^e^
Catheterization time (s), median [IQR]	45 [30, 92]	35 [27, 60]	-7 (-15, -2)	0.008^e^
Overall complications at first chosen radial artery, N (%)	35 (31.5%)	11 (9.9%)	0.31 (0.17, 0.59)	< 0.001^d^
Vasospasm, N (%)	5 (4.5%)	0 (0%)	N/A	0.060^f^
Hematoma, N (%)	34 (30.6%)	11 (9.9%)	0.32 (0.17, 0.61)	< 0.001^d^
Artery thrombosis, N (%)	0 (0%)	0 (0%)	N/A	N/A
Catheter malfunction, N (%)	1 (0.9%)	2 (1.8%)	2.00 (0.18, 21.7)	1.000^f^
The first chosen radial artery				
Diameter after cannulation (mm), median [IQR]	2.0 [1.8, 2.2]	2.1 [1.8, 2.4]	0.1 (0, 0.3)	0.008^e^
Depth after cannulation (mm), median [IQR]	2.1 [1.6, 2.9]	2.1 [1.5, 2.9]	0 (-0.3, 0.2)	0.666^e^

Continuous variables were expressed as means ± standard deviation, or median [IQR]. Qualitative variables were expressed as n (proportion%)

N/A = not applicable; CI = confidence interval; IQR = interquartile range

*d* Chi-square test. *e* Mann-Whitney U test. *f* Fisher exact test

### Secondary outcomes

Hand-eye coordination showed significant improvements in the smart glasses group, including: fewer number of head rotations compared to the ultrasound group (0 [0, 0] vs. 3 [2, 6]; *P* < 0.001; difference in medians − 3; 95% CI (-3, -3)); fewer number of ultrasound probe repositioning than ultrasound group (0 [0, 0] vs. 0 [0, 1]; *P* < 0.001; difference in medians was 0; 95% CI (0, 0)); fewer number of needle redirections than that in the ultrasound group (0 [0, 1] vs. 1 [0, 3]; *P* < 0.001; difference in medians − 1; 95% CI (-1, 0); Table 2).

**Fig. 4:**
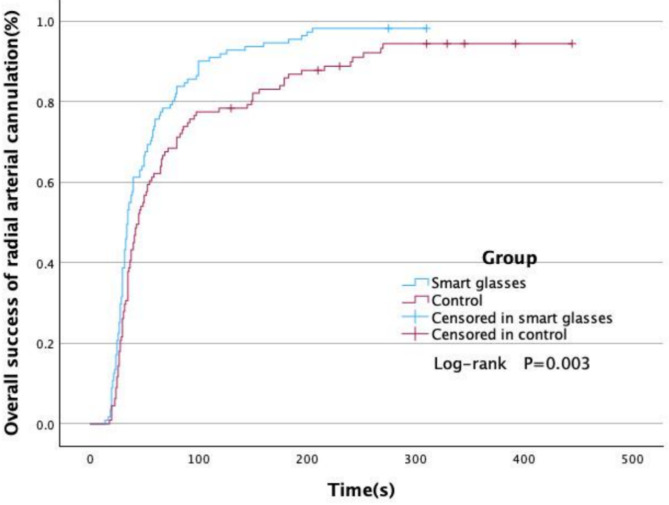
Kaplan–Meier estimates for the overall procedure time to successful cannulation radial artery (smart glasses vs. control group, *P* = 0.003)

The overall success rate of radial artery puncture was slightly higher in the smart glasses group compared to the ultrasound group (98.2% [109/111] vs. 92.8% [103/111]); however, this difference was not statistically significant (*P* = 0.052; RR, 1.06; 95% CI (1.00, 1.12)). The smart glasses group required fewer overall number of attempts than that in the ultrasound group (1 [1, 1] vs. 1 [1, 2]; *P* = 0.002; difference in medians 0; 95% CI (0, 0)). Ultrasonic localization time was shorter in the smart glasses group than in the ultrasound group (7 s [5, 10 s] vs. 10 s [6, 15 s]; *P* < 0.001; difference in medians − 2: 95% CI (-3, -1)). Catheterization time was reduced in the smart glasses group in comparison to the ultrasound group (35 s [27, 60 s] vs. 45 s [30, 92 s]; *P* = 0.008; difference in medians − 7; 95% CI (-15, -2); Table 2). Based on Kaplan-Meier analysis, the overall procedural duration from the successful insertion of the chosen radial artery was found to be shorter in the smart glasses group in comparison to the ultrasound group (*P* = 0.003; Fig. 4).

The total complication rate in the smart glasses group was less than that observed in the ultrasound group (9.9% [11/111] vs. 31.5% [35/111]); *P* < 0.001; RR, 0.31; 95% CI (0.17, 0.59)), including vasospasm (0% [0/111] vs. 4.5% [5/111]; *P* = 0.060); hematoma (9.9% [11/111] vs. 30.6% [34/111]; *P* < 0.001; RR, 0.32; 95% CI (0.17, 0.61)). We didn’t find significant difference for catheter malfunction between the smart glasses group and the ultrasound group (1.8% [2/111] vs. 0.9% [1/111]; *P* = 1.000; RR, 2.00; 95% CI (0.18, 21.7)). There was no statistically significant difference in radial artery depth after puncture between two groups. Larger diameter after intubation in the smart glasses group compared to that in the control group (2.1 mm [1.8, 2.4 mm] vs. 2.0 mm [1.8, 2.2 mm]; *P* = 0.008; difference in medians 0.1: 95% CI (0, 0.3); Table 2). However, a difference of 0.1 mm did not appear to hold significant clinical relevance.

The proportion of operators reporting no pain in ergonomic musculoskeletal fatigue scores was higher in the smart glasses group (81.1% [90/111] vs. 48.6% [54/111]; *P* < 0.001; RR, 1.67; 95%CI (1.35, 2.06)). Conversely, a lower percentage of operators in the smart glasses group reported mild pain in ergonomic scores (18.9% [21/111] vs. 51.4% [57/111]; *P* < 0.001; RR, 0.37; 95% CI (0.24, 0.56)). The proportion of satisfaction scores between 41 and 60 points was lower in the smart glasses group (0.9% [1/111] vs. 6.3% [7/111]; *P* = 0.072; RR, 0.14; 95% CI (0.02, 1.14)), which was not statistically significant. However, the proportion of satisfaction scores between 61 and 80 points was significantly lower in the smart glasses group (9.9% [11/111] vs. 24.3% [27/111]; *P* = 0.004; RR, 0.41; 95% CI (0.21, 0.78)). The proportion of operators reporting positive satisfaction scores between 81 and 100 points was higher in the smart glasses group (89.2% [99/111] vs. 69.4% [77/111]; *P* < 0.001; RR, 1.29; 95% CI (1.12, 1.48)). Regarding the difficulty levels of arterial punctures, the smart glasses group had a higher proportion of easy-level difficulties compared to the control group (53.2% [59/111] vs. 24.3% [27/111]; *P* < 0.001; RR, 2.19; 95% CI (1.51, 3.17)). Conversely, the smart glasses group had lower proportions of moderate difficulty (44.1% [49/111] vs. 62.2% [69/111]; *P* = 0.007; RR, 0.71; 95% CI (0.55, 0.92)) and difficult difficulty levels (2.7% [3/111] vs. 13.5% [15/111]; *P* = 0.003; RR, 0.20; 95% CI (0.06, 0.67); Table 3).

**Table 3:** Operator’ Questionnaire score between Control Group and Smart Glasses Group

Variables	Control (*n* = 111)	Smart Glasses (*n* = 111)	95% CI of Relative Risk or Median Difference	*P* value
Operators’ ergonomic score, N (%)				< 0.001^d^
No pain, N (%)	54 (48.6%)	90 (81.1%)	1.67 (1.35, 2.06)	
Mild pain, N (%)	57 (51.4%)	21 (18.9%)	0.37 (0.24, 0.56)	
Moderate-Severe pain, N (%)	0 (0%)	0 (0%)	N/A	N/A
Operators’ satisfaction score (0–100), N (%)				< 0.001^f^
1(0–40)	0 (0%)	0 (0%)	N/A	N/A
2(41–60)	7 (6.3%)	1 (0.9%)	0.14 (0.02, 1.14)	0.072^d^
3(61–80)	27 (24.3%)	11 (9.9%)	0.41 (0.21, 0.78)	0.004^d^
4(81–100)	77 (69.4%)	99 (89.2%)	1.29 (1.12, 1.48)	< 0.001^d^
Level of difficulty, N (%)				< 0.001^d^
Easy, N (%)	27 (24.3%)	59 (53.2%)	2.19 (1.51, 3.17)	< 0.001^d^
Moderate, N (%)	69 (62.2%)	49 (44.1%)	0.71 (0.55, 0.92)	0.007^d^
Difficult, N (%)	15 (13.5%)	3 (2.7%)	0.20 (0.06, 0.67)	0.003^d^

Qualitative variables were expressed as n (%)

N/A = not applicable

*d* Chi-square test

*f* Fisher exact test

## Discussion

The present study demonstrates that the integration of smart glasses with ultrasound significantly increases the first puncture success rate and improves hand-eye coordination compared to using ultrasound guidance alone for radial artery catheterization. Furthermore, the use of smart glasses was linked to a reduction in the overall number of attempts, catheterization time, and complication rates. These findings are consistent with those of Jang et al. [16], who reported that smart glasses improved the first attempt successful rate. Notably, our study included adults, and to standardize the operation, we unified the placement position of ultrasound machine and the operator’s sitting position, using high-frequency linear array probes to perform radial artery puncture and catheter insertion in SA-OOP approach [22]. Since there was no ultrasound position and operation mode tailored to each operator’s personal preferences, this may have contributed to the slightly lower-than-expected success rates in both the smart glasses and control groups. In the present study, the first puncture success rate and hand-eye coordination for radial artery cannulation were improved because the operator’s simultaneous gaze on the ultrasound screen and the surgical site during radial artery cannulation using the smart glasses allowed for the timely detection of the needle tail blood return. The higher success rate of first catheter insertion improved the overall success rate and reduced the overall number of attempts, catheter insertion time, and overall complication rate.

During radial artery catheterization, smart glasses improve ergonomic satisfaction and reduce procedure difficulty by minimizing the need for repeated head, neck, and eye motions between the ultrasound display and the surgical area [16, 18]. By comparison, operators in the control group were required to glance sideways at the ultrasound screen several times throughout the procedure before looking down to check for smooth blood return to the end of the needle at the puncture site [13]. In the study by Tolu Set al. [23], 69.1% of the respondents assessed the ergonomic conditions in the operating room as being below acceptable standards. In addition, the neck and low back region were the most reported body part, in the operating room. According to the study Fouad AM et al. [24], specific interventions must be implemented to address these ergonomic risk factors to improve the overall health of anesthesiologists and guarantee the safe administration of anesthesia services. Although it may seem difficult to provide a neutral working posture for anesthesiologists, medical devices can be evaluated based on ergonomic concepts and differentiated in terms of shape and details [23]. In clinical settings, the success rate of intraoperative emergency vascular catheterization was lower than that of elective surgery during anesthesia induction, more frequent instances of skin punctures or needle redirection not only heighten patient discomfort and anxiety but also amplify stress levels among physicians [25]. Considering these benefits, smart glasses could prove particularly valuable during emergency scenarios, especially when integrated with tools like wireless ultrasound devices [15].

There are several notable strengths worth mentioning. First, to the best of our knowledge, this study is the first attempt to use smart glasses combined with ultrasound for radial arterial catheterization in China. Second, this was the first study to objectively evaluate hand-eye coordination abilities, including head rotations, ultrasound probe repositioning, and the number of needle redirections. This provides valuable insights into future research related to hand-eye coordination skills. Third, the radial artery puncture was conducted using the short-axis plane, which is more commonly used in clinical practice.

However, there were some disadvantages in smart glasses. First, Smart glasses have a certain weight, and long-term use may cause nose or ear discomfort and smart glasses related ocular fatigue, especially for those who wearing glasses [26]. The findings reported by Kim et al. [27] could assist developers and researchers in designing future smart glasses that are not only more comfortable but also more effective in their intended applications. Second, wireless technology of the smart glasses in this study can’t connected with Mindray Ultrasound, and we employed a high-definition multimedia interface cable in conjunction with a digital visual interface, which limited the operator’s freedom to a certain extent [16]. Wireless technology could help operators use limited regional block areas in a more ergonomic approach [12]. However, the wireless connection to the video feed must account for potential delays in image transmission, as this lag may introduce additional artifacts and interference during the puncture and tube placement procedure. Third, if head-mounted displays are intended to be shared, nursing must establish specific infection prevention and control protocols for these devices [28]. One of the limitations of smart glasses is the weight of device, limitations of wireless connection and complexity of workflow in perioperative care. Zhang Z et al. [29] recommend conducting additional user-focused studies to better grasp the needs and perceptions of medical professionals regarding the design of smart glass technology. Moreover, investigating its effects on workflow in intricate care environments would be valuable.

Several limitations in our study should also be noted. First, the operators had knowledge of which group participants were assigned to, and non-blindness may lead to skewed assessments of ergonomics and satisfaction ratings. Second, the study included adult patients undergoing elective surgery, so the impact of smart glasses on time-constrained emergency surgery or under environmental constraints is unknown. Third, this study was conducted by clinicians experienced in using ultrasound techniques for vascular catheterization. As with any technical procedure, the proficiency and expertise of the operator are crucial factors, so the effect of smart glasses on operators with little or no experience is unknown [6, 11]. Fourth, the operator did not solicit or record patient feedback during the radial artery puncture procedure. Fifth, the sample size was not specifically designed to assess complication rates, so the conclusions regarding the study’s impact on complications should be interpreted with caution. Lastly, secondary outcomes, such as radial artery diameter and depth as well as surgery-related complications, were assessed by ultrasound, which are operator dependent and subject to interpretation errors [16].

## Conclusion

Our research indicates that utilizing smart glasses not only increases the success rate of the initial puncture but also improves hand-eye coordination during radial artery catheterization, in comparison to relying solely on ultrasound guidance. Future directions could involve integrating wireless technology, developing anatomical overlays for wearable devices, and implementing positional tracking guidance systems. These advancements aim to further enhance patient safety and comfort across various clinical procedures [12, 13].

## Data Availability

No datasets were generated or analysed during the current study.

## Abbreviations

SA-OOP: Short-axis out-of-plane
LA-IP: Long-axis in-plane
DNTP: Dynamic Needle Tip Positioning
ASA: American Society of Anesthesiologists
ECG: Electrocardiogram
VAS: Visual Analogue Scale
BMI: Body mass index
